# Interpol review of detection of AI-generated image and video deepfakes, 2022-2025

**DOI:** 10.1016/j.fsisyn.2026.100704

**Published:** 2026-06-13

**Authors:** Stijn van Lierop, Ivona Najdenkoska, Marcel Worring, Zeno Geradts

**Affiliations:** a Netherlands Forensic Institute, Laan van Ypenburg 6, The Hague, 2497GB, the Netherlands; b University of Amsterdam, Science Park 900, Amsterdam, 1098XH, the Netherlands

**Keywords:** Deepfake detection, Synthetic media, Generative AI, Image analysis, Video analysis, Authenticity of digital evidence, Forensic science

## Abstract

In recent years, there has been tremendous progress in the generation of deepfakes and synthetic media. In this systematic review, we will, from a forensic perspective, discuss the latest developments in AI generation of deepfake images and videos and methods for detecting them. We analyzed more than 400 studies published between 2022 and 2025, specifically addressing the generation and detection of AI-generated deepfakes as opposed to manipulation of existing media. We provide an overview of the developments in various generative architectures such as GANs, diffusion models, and autoregressive models, highlighting progress in visual fidelity, user control, content consistency, and computational efficiency. In addition, we outline the most common designs of detection methods, looking at various types of features that are used for detection. We conclude that innovations are mainly centered on addressing the challenges of generalizability to unseen generators and robustness against common perturbations and adversarial attacks. However, the inconsistent use of the evaluation datasets makes it difficult to compare the methods. Our review has identified several directions for future research, such as making methods more directly applicable to forensic practice by incorporating forensically relevant benchmark datasets, paying more attention to explainability, and embedding detection methods in an evidence evaluation framework.

## 1 Introduction

Deepfakes are digital media created or modified using artificial intelligence (AI), depicting an inauthentic (or fake) representation of events. Although deepfakes and synthetic media are increasingly used for legitimate purposes in, for instance, entertainment, art, education, and gaming, they also facilitate a range of criminal activities such as extortion, fraud, sexual offenses, and defamation [1]. In addition, the threat of deepfakes raises questions about the authenticity of digital media evidence such as photos, videos, and audio. Without methods to verify the authenticity of such media, there is a serious risk that fake evidence is accepted in court or authentic evidence is dismissed as fake, possibly leading to miscarriages of justice. Reliable detection methods for deepfakes and synthetic media are vital to uphold the credibility of digital media evidence in court.

**Fig. 1:**
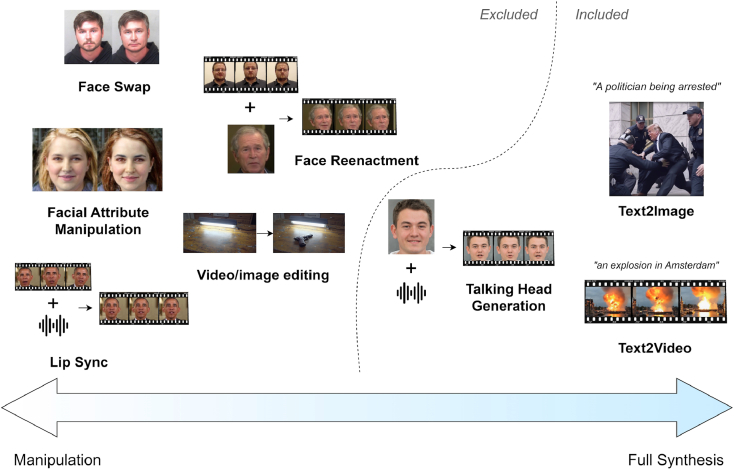
Many types of deepfakes exist, which can range from small manipulations (e.g. facial attribute manipulation) to the synthesis of entire videos or photos. This review focuses on the types of deepfakes that lean more towards full synthesis (i.e. where at least one modality is fully AI-generated).

Many types of deepfakes can be created, ranging from small manipulations in an image to entirely synthesized videos from a single text prompt (Fig. 1). In recent years, improvements in generative AI models have made it possible to synthesize media from scratch, possibly conditioned on, for instance, a text prompt or an image [[2], [3], [4]]. Many commercial tools have also started making the technology available to the general public [[5], [6], [7], [8]]. The distinction between manipulated and fully AI-generated deepfakes is not always well defined, and the boundary between them can best be regarded as a continuous scale, rather than a hard line.

The detection of deepfakes has been gaining attention in academia, with more papers being published on the topic every year [9]. Consequently, quite some reviews have been conducted to provide an overview of the literature on deepfake detection methods [[9], [10], [11], [12], [13]]. Traditionally, deepfake detection methods have focused more on detecting manipulations in existing media, such as face swaps, face reenactment, facial attribute manipulation, and lip syncing [[14], [15], [16]]. However, AI-generated synthetic media and manipulated media have been shown to leave different traces [17], are created using different architectures, and pose different challenges in terms of detection. Therefore, the topic of AI-generated media requires its own review as it involves different innovations, challenges, and possibilities for further research. To the best of our knowledge, no systematic review has yet been published that specifically focuses on AI-generated media.

The application of deepfake detection methods within forensic practice requires the consideration of specific circumstances. For instance, the explainability and interpretability of methods are of vital importance to ensure experts can rely on detection methods for their conclusions in court. Furthermore, forensic digital investigations are usually performed after the fact, making any proactive defense methods, like watermarking, that might be useful in other cases, less widely applicable for the forensic practitioner. In addition, the quality of materials is often suboptimal within a forensic context, for instance, the material often has low resolution and poor lighting. Methods designed for the forensic context have different requirements, which are often disregarded by reviews not focused on the forensic application, consequently leading to relevant research gaps being overlooked.

The goal of this review is to provide an overview of the developments in the generation and detection of AI-generated photos and videos from the last couple of years, specifically focusing on the implications for the forensic practitioner. We focus on AI-generated media where at least one modality (image or video) is completely synthesized (Fig. 1). Because generation and detection methods are constantly challenging each other, we believe it is important to look at the developments in both areas in order to get an idea of the synchronization between the fields. However, we will only discuss generation methods with the ultimate goal of detection in mind, focusing on current and future model capabilities. In addition, we limit our scope to detectors that can be used for forensic analyses after the fact, hence methods designed for active defense or live detection are not considered. As the field is moving rapidly and quite some papers are published every year, we specifically focus on publications from the last three years, that is publications from January 1st 2022 until June 1st 2025.

Overview of the paper: To understand current generative model capabilities, we will first discuss the various generative architectures by which fully synthetic photos and videos can be generated in combination with the latest developments in the field in section 3. Subsequently, we will discuss the main approaches of deepfake detection methods in section 4, after which sections 5and6 will go into the evaluation and explainability of these detection methods. The review will conclude with a discussion of open challenges regarding the detection of deepfakes as well as several promising research directions relevant for digital forensic science.

## 2 Methodology

**Fig. 2:**
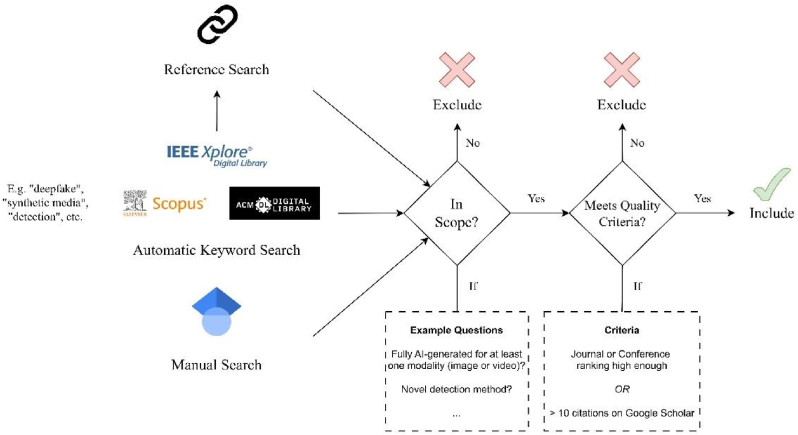
The abstract literature selection process. Sources were retrieved through advanced keyword searches in several databases and manual searches on Google Scholar. In addition, references from the retrieved sources were checked. Retrieved articles were first screened for relevance and, if in scope, their quality was assessed according to specific criteria. Articles meeting both relevance and quality criteria were included in the review.

The complete methodology for selecting literature is visualized in Fig. 2. We performed a systematic literature search using an advanced keyword query on the Scopus,^1^ IEEE Digital Library,^2^ and ACM Digital Library^3^databases. The queries used for each library can be found in Appendix A. We filtered results to include only scientific articles written in English that were published between January 1st, 2022, and June 1st, 2025 and we removed duplicates retrieved from multiple databases. In order to get as complete a picture as possible, we manually retrieved papers from Google Scholar, and references from retrieved papers were also included if they met the relevance and quality criteria specified below. Although we cannot guarantee that we have found all relevant papers in the literature, we do believe that we can provide a good picture of the current state of the field with this strategy.

Subsequently, we evaluated all articles on relevance. Deepfake detection articles were included if they presented a novel method that was evaluated on a dataset containing at least some AI-generated images or videos. Similarly, deepfake generation articles were included if they presented a novel method that would have the capacity to generate fully synthetic images or videos. Detection or generation approaches from completely different domains, such as medical imaging, live-monitoring approaches, and proactive deepfake defense methods such as watermarking were excluded. Comparative studies on detection or generation methods were included as long as they presented novel experimental results.

We then assessed the relevance of all articles within the field in several steps. Here we used a similar approach to Kombrink et al. [18]. An article was included in the review if it met at least one of the following three criteria:

1. Any article was included if it came from a source with a high enough ranking. For journals, the Scimago Journal and Country Rank^4^ was used. This ranking system uses quartiles within specific fields. Any journal within quartile Q1, Q2, or Q3 was deemed of high enough quality and would meet this criterion.

For conferences, no single ranking was used but a combination of five ranking systems. If more than half of the scores given to a conference were satisfactory, the conference would meet this criterion. The following rankings were used for this:

a) ERA (2010)^5^: scores range from A (highest) to C (lowest); Conferences that scored an A or B were included in the review.
b) Qualis (2012)^5^: possible scores from highest to lowest are A1, A2, B1, B2, B3, B4 or B5; Conferences that scored an A1, A2, B1, B2 or B3 were included in the review.
c) CORE obtained from GII-GRIN-SCIE (GGS) conference rating^6^: possible scores from highest to lowest are A++, A+, A, A-, B, Band C; Conferences that scored an A++, A+, A, A-, B, B- were included in the review.
d) LiveSHINE obtained from GII-GRIN-SCIE (GGS) conference rating^6^: possible scores are from highest to lowest A++, A+, A, A-, B, B- and C; Conferences that scored an A++, A+, A, A-, B, B were included in the review.
e) Microsoft Academic obtained from GII-GRIN-SCIE (GGS) conference rating^6^: possible scores from highest to lowest are A++, A+, A, A-, B, B- and C; Conferences that scored an A++, A+, A, A-, B, B- were included in the review.
2. Any article that was cited more than 10 times per year on average, according to the number of citations on Google Scholar, was included in the review. This criterion was included to ensure that highly influential papers that were published in sources of lesser quality could still be included. While we are aware that the number of citations on Google Scholar might not always be entirely accurate, it was the only citation count available for all papers and therefore was the only source that allowed for equal comparison between different papers.

## 3 Synthetic media generation

Generative models have revolutionized the field of computer vision by enabling the synthesis of photorealistic images and coherent videos from a wide range of data distributions. In this section, we will introduce the basics of how these generative models work. We will subsequently discuss the latest developments in the field of media synthesis, also in light of the implications for forensic practice.

### 3.1 Generative architectures

Various families of generative architectures have been developed for the generation of images and videos, each with their strengths and limitations. This section provides an overview of the main types of generative architectures that are currently state of the art (SOTA) for image and video generation, namely autoregressive models (ARs), generative adversarial networks (GANs) and diffusion models (DMs) as well as hybrid approaches. Although other families, such as normalizing flows and variational autoencoders exist, we will restrict ourselves to a more detailed discussion of the generative model families that are most relevant for current SOTA generators.

#### 3.1.1 Autoregressive models

**Fig. 3:**
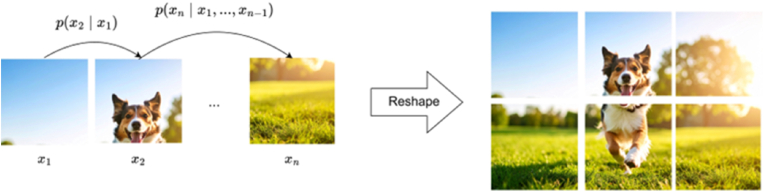
A simplified representation of the autoregressive image generation process. Each image token $x_{i}$ is sequentially generated conditioned on all previously generated tokens. When reshaped, all predicted tokens together form an image.

Autoregressive transformer models generate data by explicitly modeling the data distribution and sampling from this distribution in a sequential way (Fig. 3). The model predicts tokens iteratively, where each prediction is conditioned on all previously generated tokens. Models like PixelRNN [19] and PixelCNN [20] use pixels as tokens, which can be quite slow for images with millions of pixels and cannot quite capture the global dependencies in the image well due to the long prediction chains. Other approaches like Vector Quantized - Variational Autoencoder (VQ-VAE) [21] therefore model the data space using a set of discrete visual tokens (a codebook) instead, resulting in better global awareness in the image and fewer computational requirements. Subsequent studies have tried to improve the selection of tokens in this codebook in various ways [22,23]. OpenAI’s DALL-E [24] showed that training AR models at scale allows for the generation of high-quality images from arbitrary text descriptions that are not present in the training data.

#### 3.1.2 Generative adversarial networks

**Fig. 4:**
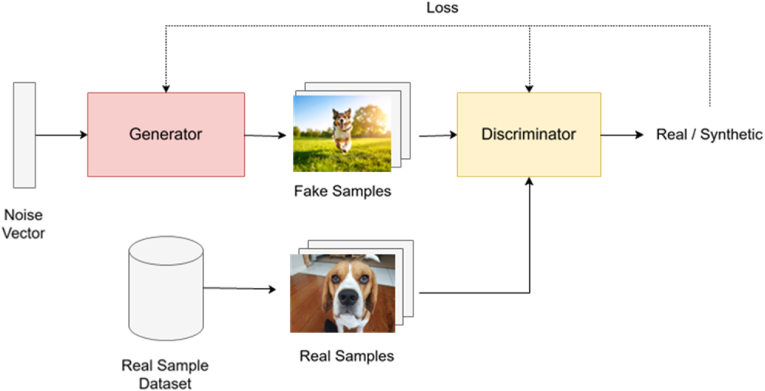
Schematic representation of a Generative Adversarial Network (GAN), illustrating the adversarial training process. The generator produces synthetic images from noise vectors, while the discriminator learns to distinguish between real samples from the dataset and fake samples generated by the generator. During training, feedback is provided to both the generator and discriminator models via loss functions to iteratively improve both networks.

GANs consist of two neural networks: a generator and a discriminator [25] (Fig. 4). The objective of the generator is to generate images that are indistinguishable from real images. It receives random noise as input, which it learns to transform into images. The goal of the discriminator is to distinguish images generated by the generator from real images. The networks are trained in an adversarial way, where the generator learns from the feedback of the discriminator. Formally, this optimization can be described as a minimax game where the following objective function is used:

$$\min_{G}\max_{D}V(D,G)=E_{x∼p_{\text{data}}(x)}[\log D(x)]+E_{z∼p_{z}(z)}[\log(1-D(G(z)))]$$

If training is successful, the generator will eventually learn to generate images that are indistinguishable from real images. However, training GANs is not trivial and can be quite challenging. If either the generator or the discriminator outperforms the other, training becomes unstable and learning will cease. Other issues, such as mode collapse or vanishing gradients may occur as well.

Many different GAN architectures have been developed to overcome the above-mentioned limitations and generate more realistic images. Deep Convolutional GAN (DCGAN) was proposed as one of the first methods to improve on training stability by making modifications to the network architecture and demonstrating that the latent space could learn semantically meaningful directions [26]. Mode collapse issues have been addressed by architectures like Wasserstein GAN (WGAN) [27] and Wasserstein GAN with gradient penalty (WGAN-GP) [28] by redefining the loss function and introducing a penalty on the gradient norm of the discriminator’s output. Progressive GAN (ProGAN) [29] introduced the concept of progressive training where training initially starts with low-resolution images and layers are incrementally added to increase resolution, which helps increase training stability. Including a minibatch standard deviation layer in the discriminator helps improve diversity in generated samples. Models like StyleGAN [30] and later StyleGAN2 [31] add increased generation control by incorporating a latent space that disentangles semantic attributes such as pose and identity. BigGAN [32] improves on visual fidelity at the cost of model size by doubling the feature maps in each layer and integrating self-attention layers from SAGAN [33] in combination with spectral normalization. GigaGAN [34] and StyleGAN-XL [35] show that leveraging large image datasets in combination with a carefully designed architecture results in SOTA image generation performance with faster, higher resolution synthesis and the possibility of various latent space editing applications such as latent interpolation, style mixing, and vector arithmetic operations. Other StyleGAN architectures incorporate further improvements such as real-time generation in StyleGAN-T [36] and resolving of texture-sticking and aliasing artifacts in StyleGAN3 [37].

#### 3.1.3 Diffusion models

**Fig. 5:**
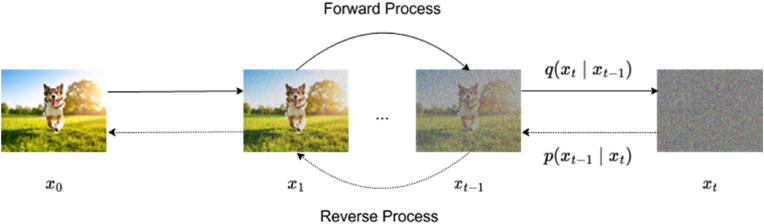
The fundamentals of a diffusion model. The forward process (top arrows) gradually adds noise to an image $x_{0}$ over multiple steps, producing increasingly noisy versions $x_{1},\ldots ,x_{t}$ until reaching pure noise. The reverse process (bottom arrows) learns to progressively denoise $x_{t}$ back to the original image $x_{0}$, modeling the conditional probability $p\left(x_{t-1}|x_{t}\right)$ at each step. By sampling noise and applying the reverse process for several time steps, an image is generated.

Diffusion Models (DMs) are a family of models that have learned to generate data from random noise using a probabilistic denoising process (Fig. 5). DMs are trained using two processes: a forward process where noise gets incrementally added to an image until pure noise remains, and a reverse process where a neural network such as a U-Net [38] is learned to denoise the image iteratively over time steps until an image emerges. DMs have demonstrated superior performance compared to other generative approaches across multiple domains and applications [[39], [40], [41]]. The formulation that most DMs are based on is Denoising Diffusion Probabilistic Models (DDPMs) [40]. DDPMs have been trained to remove noise in a fixed number of discrete timesteps. During the forward process in each timestep, some random noise is added where the output timestep *t* is used as input for timestep *t*+1. However, because of this random noise that is added at every step, the sampling process is non-deterministic and samples can only be generated in the reverse process by iteratively denoising over all timesteps separately. Diffusion Deterministic Implicit Model (DDIM) [41] addresses this problem by making the sampling process deterministic using a fixed noise schedule instead of random noise during the forward process. This allows DDIM to skip sampling steps, resulting in much faster sample generation compared to DDPM.

The reverse process, going from timestep *t* to timestep *t*−1 can also be conditioned on additional input information such as text or images. A method like classifier-free guidance [42] can be used to incorporate input information from text or images to guide the diffusion process in a certain direction. During training, a DM learns to generate images with and without conditioning information, such as a text or an image, by using semantic knowledge embedded in a vision language model like CLIP. During sampling, the difference between the two is used to guide the diffusion process. Models do not exclusively have to be conditional or unconditional, as is illustrated by models like GLIDE [43] that allow for both conditional and unconditional image generation.

Many subsequent architectures are based on DDPM and DDIM. Latent diffusion models (LDMs) [2], such as Stable Diffusion (SD), move the denoising process from the pixel domain to a latent space, thereby addressing computational limitations in the generation of high-resolution images. Stable Diffusion XL (SDXL) uses a three times larger U-Net, increasing visual fidelity at the cost of computational complexity [44]. Ablated Diffusion Model (ADM) improves on sampling speed by using a motion pattern estimator to predict prior noise distributions of trajectories [39]. DeepFloyd-IF [45] moves the denoising process back to pixel space by utilizing an adapted U-Net architecture in combination with super-resolution layers for high-resolution output. Other innovations, such as Spatial-Frequency U-Net (SFUNet) [46], have explored diffusion modeling in wavelet domains by combining spatial and frequency information to enhance generation quality and efficiency.

In addition to DDPMs, alternative formulations of DMs have emerged as well. Score matching with Langevin dynamics (SMLD) estimates the score (i.e. the gradient of the log probability density with respect to the data) at each noise scale and uses this to guide sampling via annealed Langevin dynamics [47]. Stochastic Differential Equations (SDEs) generalize this framework by modeling the diffusion process through a continuous-time framework rather than discrete steps [48]. While these approaches allow for more complex architectures and more flexibility, DDPMs are more common in practice since they are easier to implement and can achieve similar or even better visual quality [49]. Rectified Flow models propose a new generative modeling paradigm where data is transformed into noise (and vice versa) along straight-line trajectories in data space, rather than the random, winding paths of traditional diffusion models [50,51]. These models have been adapted for use in the latent space [52] and with AR models [53] as well.

DMs have also been extended to the video domain. Many of these models build directly on pre-trained text-to-image models by adding a temporal component, eliminating the need to learn visual concepts from scratch. This strategy accelerates training and helps the model focus more on learning realistic motion and temporal dynamics [54,55]. Video Diffusion Model (VDM) uses a pretrained image diffusion model, but incorporates temporal attention and a 3D U-Net architecture to generate temporally consistent videos [56]. However, temporal attention can also be incorporated in other ways. For instance, by implementing cross-frame attention [3]. Latent video diffusion models (LVDMs) move the denoising process to the latent space, allowing more efficient computations for the generation of longer and higher quality videos [[57], [58], [59], [60]].

#### 3.1.4 Hybrid approaches

Hybrid architectures combine ideas of two or more model families. Notable examples are Diffusion-GAN [61], StyleSwin [62] and taming transformers [63]. Advantages of these approaches are that they can combine the advantages of multiple model families in a single model, often achieving fast, high-fidelity, and diverse generation output. A widely used hybrid architecture is the Diffusion Transformer (DiT) [64], which replaces the traditional U-Net backbone in DMs with a transformer architecture. A well-known DiT model is DALL-E 3 [65], which uses an LLM-based text encoder in combination with a hybrid diffusion approach in both latent as well as pixelspace. Other recent models like DiffiT [66], PixArt-*α* [67], HDiT [68] and FLUX1 [69] further demonstrate the added benefits of combining the high output quality from diffusion frameworks with the scalability advantages of AR transformers.

#### 3.1.5 Other approaches

Some other approaches commonly seen in the deepfake detection literature are perceptual loss models and low-level vision models. Perceptual loss models like IMLE [70] and CRN [71] make use of perceptual loss functions. Instead of measuring error by comparing the pixel values of two images directly, these loss functions measure error by comparing the embeddings of two images extracted from a neural network. As a result, these high-level models tend to pay more attention to visual similarity, rather than pixel-by-pixel accuracy.

In contrast, low-level vision models such as SAN [72] and SITD [73], focus on extracting and processing basic features from images like textures, edges, colors, and motion. These models use local operations that are closely related to the raw pixel values and differ in this sense from high-level models, which deal with more abstract and semantic concepts such as object recognition or scene understanding.

#### 3.1.6 Commercial models

In recent years, many commercial models and applications have become available that make the technology for generating images and videos accessible to the general public. Tools like Midjourney [6], Google Imagegen [74] and OpenAI GPT-4o [75] for images, and Sora [5], Kling AI [7], Veo3 [8], Imagen [76], Runway Gen-4 [77], Luma Dreammachine [78] and Meta’s MovieGen [79] for videos are capable of generating SOTA quality media. Many of these models build on existing technologies, but often implement new innovations. Since they are often not published in the scientific literature, not all details about the inner workings of these models and their development are known. However, information from technical reports, behavior of these models, and detection studies deliver useful insights about how these models work.

We know that many commercial image and video generator models are built on DM or DiT architectures. The architecture of Midjourney is proprietary, although detectors trained on SD1.4 have shown good generalization performance on the detection of images generated by Midjourney, suggesting that Midjourney is most likely a DM [80]. Google’s Imagegen and Runway both use a cascaded diffusion process (similar to GLIDE). OpenAI’s GPT-4o makes use of an autoregressive multimodal large language model to generate images, making it capable of high-fidelity image generation with consistent content and image editing capabilities.

Many companies try to prevent abuse of their models by enforcing content violation policies that prohibit the generation of harmful content. In addition, models such as Google’s Imagen watermark generated media with an invisible watermark so it can be identified as AI-generated out-of-context as well [81]. Although these measures can be helpful with verification of media and can help protect against the generation of harmful content, studies have shown that adversarial techniques can circumvent content violation policies [82], and watermarks can be removed [83]. Only relying on these technologies for detection is therefore not enough. We will discuss other detection methods in section 4.

### 3.2 Recent developments

**Fig. 6:**
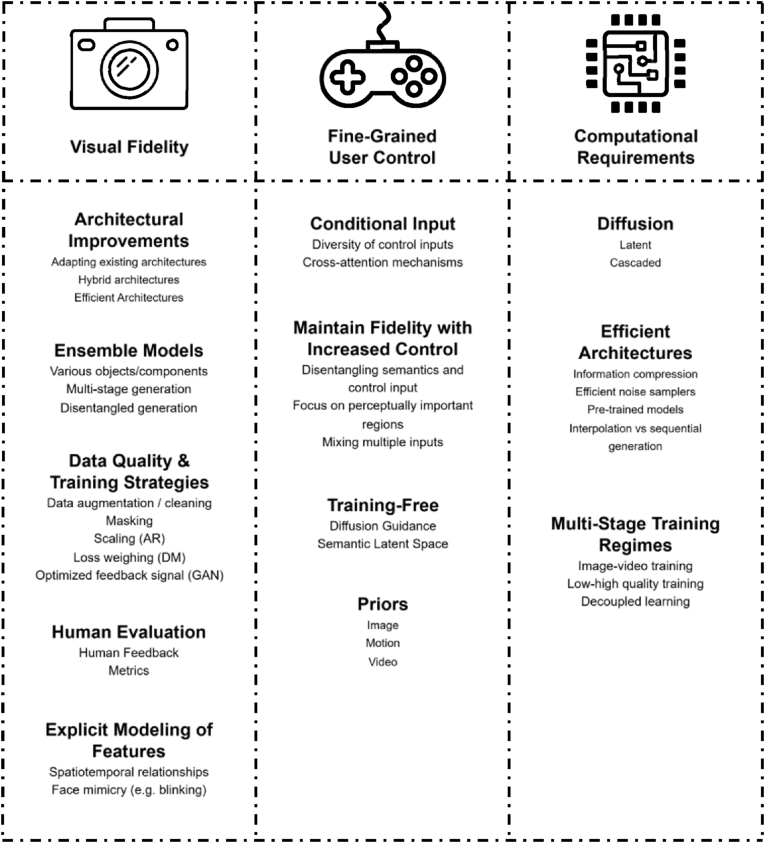
An overview of the main focus areas of image and video generators in the literature. Three main trends can be observed regarding visual fidelity, fine-grained user control and computational requirements.

We identified several major trends in the development of generative models: improved visual fidelity, fine-grained user control and increased computational efficiency (Fig. 6). We will discuss each of these in more detail in the sections below. It is important to emphasize that these trends are very much connected to each other. More fine-grained control may lead to higher fidelity output for instance.

#### 3.2.1 Visual fidelity

Despite significant advances in the photorealism of generative models, ongoing research continues to address challenges in generation quality, particularly for complex or underexplored domains such as human faces [84], satellite imagery [85,86], and less perceptible image properties like its frequency spectrum [87,88]. In practice, visual fidelity is most often measured using metrics like Fréchet inception distance (FID) [89], peak signal to noise ratio (PSNR) and structural similarity index measure (SSIM) [90], besides assessing visual quality subjectively. Nevertheless, these metrics may not always agree with human evaluation and approaches have therefore underscored the role of humans in evaluating the generation quality of generative models [91,92]. As a result, datasets for human evaluation [93] and new metrics that capture the human preference have been introduced [94].

Many architectural improvements have been proposed to make the generation of higher fidelity content possible. For instance, by adapting CycleGAN with an attention mechanism and adaptive residual blocks [95] or integrating autoencoder-based attention modules in StyleGAN [96]. Recent innovations in AR models have explored continuous autoregressive generation without discrete tokens, thereby mitigating information loss during the quantization step, resulting in high fidelity output with more detail [97]. Smart training strategies and adapted loss functions have resulted in improved performance as well with various architectures [98,99]. Increased diversity without loss of fidelity has also been a focus area, in particular for DMs. Proposed solutions have been to explicitly model image representations [100] or to incorporate autoregressive latent modeling in the diffusion process [101]. Relational understanding has been identified as critical for realistic image synthesis as well. Ye et al. [102] developed RaT2IGen, which employs learnable prompts and a relation-aware cross-attention module within the diffusion U-Net to enhance relational reasoning during generation. Lastly, more efficient architectures may allow models to contain more parameters, which can lead to higher quality generations [103].

Another strategy to increase visual fidelity is to combine multiple generators, either at an abstract level during the diffusion process [104] or at the semantic level where different models generate different components of the output, such as body parts, objects, persons or scenes [95,105,106]. Disentangling motion from appearance has also resulted in improved spatiotemporal quality [107]. While such ensembles can improve visual fidelity, achieving both diversity and consistency across generated outputs remains challenging. Alternatively, multi-stage generation strategies refine images iteratively from coarse to fine structures [108].

Besides model architecture, the importance of data quality and training strategies for achieving high-fidelity results has also been stressed. Such strategies may include a combination of data augmentation techniques, label smoothing, careful finetuning, cleaning and captioning data, and selecting a smart loss function [4,109,110]. Employing masking during training has also been shown to increase visual fidelity and generalization to new domains not seen during training [111,112]. For ARs, scaling has been proven an effective strategy [24,113], while prioritizing the losses of medium noise levels during training has resulted in improved generation quality for DMs [114]. For GANs, several approaches have been proposed to optimize the feedback signal provided to the generator during training by deploying an ensemble of discriminators to decouple the aspects of fidelity and coherence with control input [115] or by passing GRADCam activation maps of the discriminator to the generator [116]. Furthermore, incorporating and refining negative prompts has also been shown to improve visual fidelity and prompt adherence [117].

For video synthesis, achieving spatiotemporal consistency is a major challenge. Explicit modeling of spatial-temporal relationships using cross-attention mechanisms has led to improvements in text-video alignment, per-frame quality, and temporal coherence [118]. Realism in talking head generation videos has been increased by designing methods to add subtle details such as eye blinking, slight head movements, more expressions of emotion and by adding motion priors that restrict the face movement space to realistic expressions [[119], [120], [121]]. Current video synthesis methods however still struggle with spatiotemporal artifacts such as texture-sticking, unnatural merging of elements, inaccurate physics as well as sudden appearances and disappearances in frame.

#### 3.2.2 Fine-grained user control

A second major challenge in the field is allowing for fine-grained user control over the generation process. While prompting strategies and LLMs can help formulate more precise instructions [122,123], the most effective approaches incorporate additional user-provided constraints. Semantic masks in particular, which represent abstract spatial labels, have been widely adopted. Many current strategies try to increase user control while preserving image fidelity and diversity in various ways, such as disentangling semantics and control input [115,124,125], focusing on perceptually important regions [126] and using input from multiple existing images [127]. Another strategy in transformer models is token-merging, which merges tokens related to the same object and its attributes, thereby leading to better prompt adherence [128].

Frameworks like GLIGEN [129] and ControlNet [130] provide an architecture to retrain existing pre-trained DMs and make them compatible with a wide variety of control inputs such as bounding boxes, keypoints and images. These frameworks have been refined further by many methods, addressing issues like control input variety [[131], [132], [133]], efficiency [134,135], responsiveness [136,137] and local influence [138,139]. This approach has been extended to autoregressive [140,141] and video synthesis models as well [[142], [143], [144], [145]].

Many other methods add user control by incorporating some custom form of a cross-attention or mutual self-attention mechanism between encodings of an input image and a reference image or text prompt [[146], [147], [148], [149], [150], [151], [152], [153], [154]]. Specific encoders can be trained to extract specific concepts from provided images so the right content is depicted in the desired way in the output, for instance, by extracting pose from one image and background from another [155].

Several approaches also enable consistent image generation in a training free manner by guiding the diffusion process in improved ways [[156], [157], [158], [159], [160]] or through the design of interpretable latent spaces [122,161,162]. An important focus in this line of research is the disentanglement of various semantic features in the latent space [163]. Furthermore, innovations such as FreeDoM have improved the conditioning process to allow for guiding across conditions [164]. Methods can also directly incorporate users into the model finetuning loop using active learning [165].

For video synthesis, many methods incorporate priors that constrain the generation space resulting in increased user control. As an added benefit, constraining the generation space, for instance, by incorporating images as spatial prior [166] or motion patterns from an input video [167,168] as temporal prior, can also result in higher fidelity generated videos compared to when only a text prior is provided. Multiple text2video approaches have tried to omit this additional input by incorporating learned motion priors [169,170], or enable video synthesis by designing a modular motion module that can be added to existing text2image models [171]. Geometrical constraints have been used to strengthen the association between different viewpoints, enabling the generation of longer videos of novel views from a single image [172]. Lastly, motion is often only roughly described by text and therefore much harder to control as opposed to spatial descriptions, which are usually more detailed. Chen et al. showed that such motion descriptions can be better incorporated by training a motion module that is added to a pretrained DM [173].

#### 3.2.3 Computational requirements

The generation of high-quality media, particularly videos, presents significant computational challenges. Many architectural improvements have been proposed to make models more efficient and generate higher resolution media [[174], [175], [176]]. Some common strategies for DMs are moving the denoising process to a latent space [4,54,85,141,177,178] or utilizing cascaded diffusion operations where denoising takes place from low to high resolutions [[179], [180], [181], [182], [183]]. Other approaches have tried to generate higher resolution media by using local instead of global attention [184] or make use of spatiotemporal compression strategies [103,178,185,186]. In addition, several publications have proposed strategies to leverage the knowledge of existing image and video generators to create new, more efficient generators, while achieving similar or even better visual fidelity [187,188].

Generator speed improvements have been obtained by designing more efficient noise samplers during the diffusion process [92,[189], [190], [191]], simplifying the diffusion score function for complex diffusion processes [175], or leveraging features from earlier pre-trained DMs [192]. Generation of longer videos has been made possible by interpolating between multiple generated frames instead of generating all frames sequentially [193] as well as integrating causal attention masks [55]. Regardless of all improvements, achieving high-resolution output in real-time is still a challenge, especially for video synthesis models.

Lastly, the lack of high-quality multimodal datasets remains a bottleneck for training robust models that can generate videos from text or images. To address this, approaches have found smart ways to work with the data that is available. Techniques such as generating pseudo-text features with CLIP [194], leveraging large-scale text-image datasets for pretraining, and subsequent unsupervised video training [4,195] have been deployed. Additional strategies include staged training regimes that first establish spatiotemporal coupling on low-quality data before refining spatial quality on high-resolution images [196], as well as decoupling semantic and visual quality learning into separate phases [197]. More efficient methods that require less training data have also been proposed [198].

## 4 Synthetic media detection

**Fig. 7:**
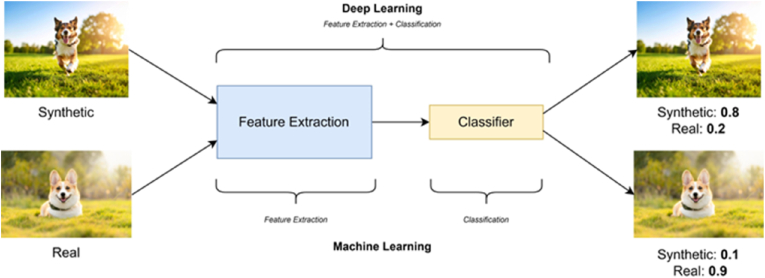
The general pipeline of a deepfake detection method. Input images undergo feature extraction, after which a classifier determines whether an image is synthetic or real based on the extracted features, often producing a score for each class. For deep learning approaches, the feature extraction and classification components are integrated in the same network, whereas for simple machine learning approaches, these components are separated.

Numerous approaches have been proposed for the detection of synthetic media, mostly aimed at synthetic images. The ideal detection method generalizes well to unseen generators, is robust to postprocessing operations like compression, and is explainable. Although the majority of these techniques incorporate feature extraction and classification components (Fig. 7), the design in which these elements are organized varies considerably. To provide a structured overview, we will therefore first examine the range of features employed in deepfake detection and what classification strategies have been used. Subsequently, we will discuss the overall architectures utilized in the literature, detailing how various features are integrated to detect synthetic media. Finally, we will address some general training methodologies and strategies designed to evade detection that have been proposed in the literature.

### 4.1 Feature extraction

**Fig. 8:**
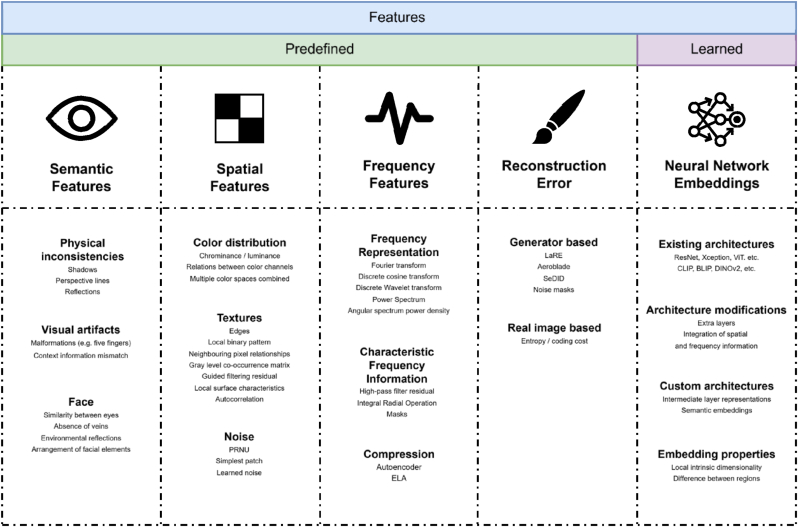
An overview of features commonly used for deepfake detection in the literature. Features can be categorized into five categories: semantic, spatial, frequency, reconstruction-based, and neural network embedding. Within each of these categories, multiple approaches can be distinguished. Green columns indicate predefined features while purple columns indicate learned features.

The features that have been used for deepfake detection in the past years are shown in Fig. 8. Features can be divided into several main categories: semantic, spatial, frequency, reconstruction error, and deep learning features. The first four categories include predefined features that are manually defined, while the fifth category includes learned features that are extracted by a trained deep learning model. All features will be discussed in more detail in the sections below.

#### 4.1.1 Semantic features

Semantic features are related to the semantic content of the image and usually depict some artifact or physical impossibility. This includes obvious artifacts such as distorted limbs or malformed faces, but also more subtle features such as inconsistent perspective lines, misaligned or inconsistent shadows, asymmetries, and arrangement of facial elements [[199], [200], [201]]. Most research on semantic features has focused on faces. The eyes in particular have been a source of many features, including the absence of veins in the sclera of the eyes [202], the shape of the pupils [203,204], similarity between the eyes [[203], [204], [205], [206]], and reflections visible in the eyes [[204], [205], [206]]. A combination of multiple features appears to result in the best overall performance [206]. An advantage of semantic features is their interpretability.

#### 4.1.2 Spatial features

Spatial features are related to the values of the pixels and their position in the image or video. The color distribution of pixels in the image has been shown to be informative, as many generators cannot accurately reproduce the natural color distribution of real images yet [207]. Many studies therefore transform images to YCyCb color space to separate the chrominance from the luminance component [207,208], but representations from multiple color spaces may also be used as input [209]. Other color-related features include the co-occurrence matrices between color channels [210] or differences between color channels [208,211,212]. While color features generally seem to be informative, there is also evidence that their efficacy diminishes significantly after images are post-processed using operations like compression [210].

Besides color, texture features may be useful as well. Such features can be extracted using Sobel filters, local binary pattern filters, Canny edge detection, or Gabor filters [[213], [214], [215], [216], [217], [218], [219], [220]]. Other methods have used texture [221] or contrast [222,223] enhancement techniques to emphasize these features rather than extract them. Features from the gray-level co-occurrence matrix such as the angular second moment (ASM), correlation, dissimilarity, entropy, energy, and inverse different moment have also been widely adopted [201,215,223,224]. Tan et al. define a feature called neighboring pixel relationships (NPR) [225] which is later used by Fu et al. as well [226] to capture upsampling artifacts in the spatial domain. Guo et al. isolate artifact traces using guided filtering [227], while Ciamarra et al. estimate surface characteristics of the face [228]. Others have found autocorrelation to be an informative feature, as synthetic images would exhibit greater self-similarity compared to natural images [229,230].

The noise profile of an image or video may be used for detection as well, as this aspect is often neglected by generative models. Several approaches extract the photo response non-uniformity (PRNU) [[231], [232], [233]] signal, which has traditionally been used in camera source identification. Chen et al. restrict themselves to the noise pattern found in the simplest patch of an image as it is less influenced by texture-rich regions [234]. Liu et al. learn noise patterns from real images and detect deviations to avoid overfitting on artifacts from specific generators [235].

For video, Wang et al. proposed to use the frequency of motion prediction modes in a video sequence. This idea is based on the observation that fake videos tend to have less fluent and more unnatural motion compared to real videos [236].

#### 4.1.3 Frequency features

Current generative models have shown deficiencies in accurately reproducing the frequency spectrum of natural images, in particular, the high frequency areas. To extract these discrepancies, images are transformed into the frequency domain using techniques such the Fourier transform [212,213,219,223,229,[237], [238], [239], [240], [241]], discrete cosine transform [229,[242], [243], [244], [245]], discrete wavelet transform [217,239,[246], [247], [248]] or fully separable wavelet transform [249]. Additional approaches utilize the power spectrum [250,251] or angular spectrum power density [230] of an image. Some methods further simplify these features by extracting only characteristic peaks with a high-pass filter [207,208,231,237,[251], [252], [253]]. Others amplify the informative parts of the frequency spectrum using learned masks [254] or an integral radial operation [238].

Compression-based methods, based on the idea that synthetic images would be easier to compress using a neural network than real images, have also been explored [255]. In addition, Error level analysis (ELA) [232], a technique that can identify JPEG compression artifacts, could be useful. Martin-Rodriguez et al. compared PRNU and ELA techniques and found that ELA features were slightly more informative compared to PRNU when used as input for a CNN classifier [232].

The main advantages of frequency features are that they are easy to extract, can be explained, and can be quite characteristic of a particular generator. This makes them potentially superior to other features for classification [229]. However, postprocessing operations such as resizing and compression can significantly alter the frequency spectrum, obfuscating or completely removing these traces in many real-world scenarios [256]. In addition, observed artifacts are not only dependent on the generator, but also very much on the dataset and details of the generator architecture, making it difficult to train detectors that generalize well [230].

#### 4.1.4 Reconstruction error

Reconstruction error is based on the idea that synthetic image generators are more adept at reconstructing artificial images as compared to natural images. Hence, by encoding and decoding an image using an autoencoder or diffusion generator and calculating the difference between the original and reconstruction, a reconstruction error can be estimated. Multiple papers have experimented with this idea using different encoding and decoding models such as Stable Diffusion [257,258], ADM [259] and LDM [260] for images and videos [261]. Recent advances calculate the reconstruction error per denoising step [262] or take the reconstruction error at all timesteps [263] to get a richer feature representation.

Several variants on the default reconstruction error approach have been proposed as well. Zhu et al. use latent DM model activations instead of the reconstruction as a feature [264]. Yang et al. leverage the susceptibility of synthetic images to noise by learning an adversarial noise mask that is added to an image [265]. After passing the image with adversarial noise through a DM, the reconstruction of distorted images should be more distorted than those of real images revealing the nature of an image. Cozzolino et al. use a model fitted on real images instead of a synthetic image model. They classify an image as synthetic if it deviates significantly from this real image model [266]. If real reference material is available, such as with faces of real people, possible deviations in generated content from the real face may also be used for detection.

#### 4.1.5 Neural network embeddings

Recent literature predominantly utilizes features extracted from (pretrained) deep learning models, commonly referred to as embeddings, for the detection of synthetic media. Deep neural networks are capable of encoding images into feature vectors and can also combine multiple features into new representations.

Many methods employ established model architectures that have been pretrained on large-scale image datasets such as ImageNet [267]. These pretrained models may be used as-is, or further fine-tuned for the task at hand. Architectural modifications are sometimes introduced, for example, by appending a classification layer. Commonly used CNN embedding networks are ResNet [17,218,224,226,227,229,238,239,242,252,262], [[268], [269], [270], [271], [272], [273], [274], [275], [276], [277], [278], [279], [280], [281], [282], [283], [284], [285], [286], [287], [288], [289], [290], [291], [292], [293], [294], [295], [296], [297]], VGG [229,283,287,292,294,[298], [299], [300]], VGGFace [294], EfficientNet [17,274,286,291,296,[301], [302], [303], [304], [305], [306], [307], [308], [309], [310]], Xception [218,269,283,284,287,291,304,311,312], DenseNet [17,220,224,274,284,286,287,292,294,296,298], GRAMNet [224,313], MISLNet [314,315], SNet [292], Inception [17,283,284,287], MesoInception [304,316], InceptionResNet [317], OctResNet [212], BNext [219], ShuffleNet [17,309], Conv-4-64 [318], JointCNN [319] and MobileNet [17,275,287,307,320].

Besides CNNs, vision transformers [17,218,221,231,274,286,292,302,305,307,[321], [322], [323], [324], [325], [326]], and variants such as Swin transformers [295,327] have also been used. A significant number of papers leverages the pre-trained CLIP model [244,248,269,272,[328], [329], [330], [331], [332], [333], [334], [335], [336], [337], [338], [339], [340], [341], [342], [343], [344]], which sets itself apart from traditional vision transformers in its contrastive language-image pretraining on a largescale image-text pairs dataset, enabling multi-modal understanding and zeroshot classification. CLIP has been used for synthetic video detection as well in combination with a DeMamba block to incorporate temporal information [345]. Other publications have utilized different multimodal modals, such as BLIP [328,[346], [347], [348]], DINOv2 [349] and ImageBind [328]. While CLIP-based feature extractors often outperform traditional CNN-based methods, no single extractor consistently achieves superior performance across all scenarios.

Existing architectures have also been modified by adding extra linear layers, dropout layers or channel attention mechanisms [209,306,350]. Other modifications include custom blocks to enable hybrid spatial and frequency feature extraction [338,351,352], isolation of particular layers for inference [277] and transformation of pretrained model features for adaptation to the deepfake detection task [341].

Custom feature encoders have been trained as well, most often inspired by the contrastive learning strategies from CLIP-like models [273,293,307,338,353,354]. Sometimes encoders are designed to produce meaningful embeddings capturing a particular feature such as high-frequency traces [355], face naturalness degree [356], non-local features [357], gradient features [358] or forgery feature types [359]. Experiments have shown that contrastively optimized encoders may not always produce features that perform better for seen generators compared to binary optimized CNNs, but there is evidence that they may generalize better to generators unseen during training [354]. Disentanglement approaches train encoders to produce two or more disentangled embeddings, often one related and one unrelated to the relevant traces for detection [295,336]. This helps reducing the influence of irrelevant information such as semantic content, and improves generalization.

The distribution of embeddings may also be informative. Local intrinsic dimensionality of embeddings has been found to contain useful information for detection [360] as well as the difference between embeddings of texture-rich and texture-poor regions [361]. LLMs have been used for deepfake detection mainly in a zero-shot fashion. GPT4V was found to perform much better compared to Gemini 1.0 when asked to classify images as real or deepfake, although performance was primarily high for fake images [362].

Many papers have also designed their own completely custom end-to-end architectures, including several CNNs [109,232,235,240,292,298,304,[352], [353], [354],^356^,^359^,[363], [364], [365], [366], [367], [368]], deep residual networks (DRN) [222], hybrid networks [369,370] and Bayesian neural networks [247]. Some combine multiple features such as multimodal features [371], frequency features [253] or various upsampled versions of the input [372]. Other approaches try to incorporate intermediate representations of the feature encoding model as features into the classification backbone [217,332] or use these for localization of forged regions [311].

Deep learning embeddings can be quickly extracted and often capture rich representations that other types of features do not. However, the features themselves are difficult to interpret, making the overall method more difficult to use in the forensic-legal framework.

### 4.2 Classification

Most methods use a machine learning or deep learning classifier. Classifiers can be trained jointly with the rest of the model or can be trained separately, for instance when pre-trained encoders are used. A relatively common practice is called linear probing, where only a linear layer with a softmax function is trained to transform an embedding or feature vector into predictions [205,206,219,220,227,231,268,277,287,289,304,307,316,323,330,332,333,335,336,340,341,348,352,353,356,370]. Other common classifiers are simple machine learning algorithms such as a support vector machine (SVM) [211,213,233,236,251,298,301,307,325,333,339], linear regression model [211], random forest [201,211,243,245,250,360], KNN [211,277,325], Gaussian Mixture Model [211,271], Naive Bayes [211,325] or XGBoost [211,373]. Deeper neural networks have been used a lot for classification as well [2,200,202,207,208,210,211,217,218,222,223,225,228,241,248,249,252,255,261,264,265,269,272,274,303], [[307], [308], [309]], [320,324,325,328,329,334,337,342,345,357,358,361,374,375].

When not using a deep learning or machine learning classifier, predictions are often based on a similarity or distance measure to a reference point or population, for instance when an extracted model’s "fingerprint" is compared to a library of references or prototypes [80,254,349]. These approaches may require calibration if a threshold is used to assign a label, or may assign the label with the highest similarity or smallest error [203,258,260,262,271,360,363,376]. Finetuned vision language models such as CLIP do not necessarily require a reference as they can directly compare the similarity of an image encoding to text encodings of the labels "real" and "fake" [335,338,346]. Instead of similarity to a particular text label, similarity to another set of images with a known label can also be used during testing [273]. Besides commonly used similarity or distance measures such as cosine similarity, custom metrics tailored to the deepfake detection task have been proposed as well [318].

### 4.3 Model design

Methods can integrate various features and classification components in many different ways. We have identified several main model designs that come back within the deepfake detection literature: models use either single features, multiple features or an ensemble of models.

#### 4.3.1 Single feature models

**Fig. 9:**
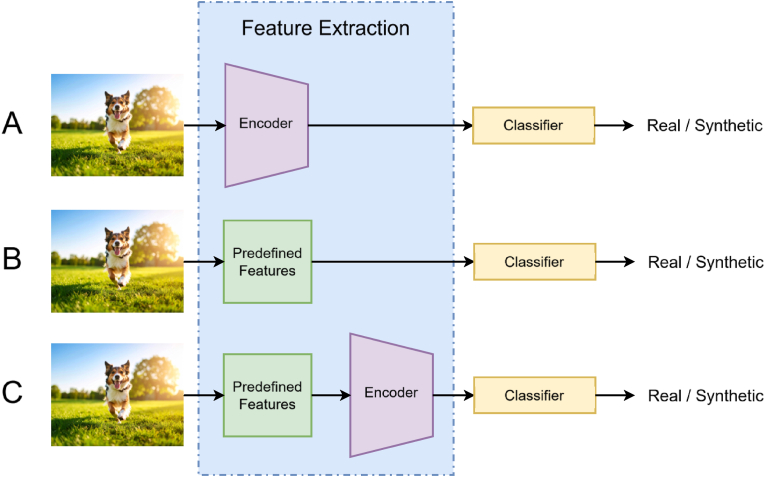
Types of single-feature models commonly used in the literature. (A) Features are extracted using an encoder and then classified. (B) Predefined features are used directly for classification. (C) Predefined features are further processed by an encoder before classification.

A large number of models only use a single feature extraction branch. These models usually have one of the three designs depicted in Fig. 9. A first design uses an encoder network to transform input images into discriminative embeddings for classification (Fig. 9A). This encoder-classifier pipeline can be trained end-to-end [[375], [376], [377]] as is done by many approaches that finetune a pre-trained model. Alternatively, the encoder can be trained separately from the classifier [264,344], which is done by many linear probing approaches.

In a second design, predefined features are extracted from an input image and passed to a classifier (Fig. 9B). These can be any features that are not embeddings such as PRNU patterns [232], facial features [200] or DCT frequency features [245]. This design usually allows for the most explainable methods, provided that a simple classifier is used.

In a third hybrid design, predefined features are extracted first after which they are encoded by an encoder model to produce embeddings for classification (Fig. 9C). Most often, this design is used by methods that pass predefined features to a neural network for encoding and classification, such as local binary pattern features that are passed to a DenseNet network [220] or neighboring pixel relationship features that are used as input for a ResNet50 model [226].

In all cases, separate models can be used for encoding and classification, but the encoder and classifier can also be jointly trained and optimized. In addition, feature extraction may involve some preprocessing or postprocessing operations such as median or bilateral filtering to remove noise [200,320] or PCA to reduce dimensionality of the feature vector [301,310].

#### 4.3.2 Multi-feature models

**Fig. 10:**
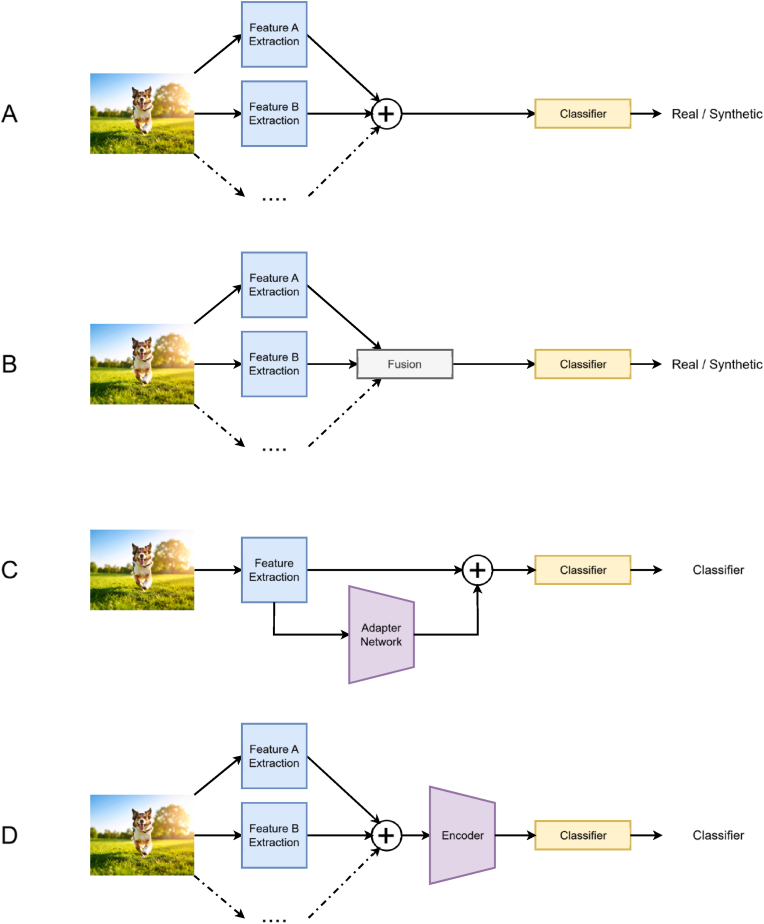
Types of multi-feature models commonly used in the literature. (A) Multiple features are extracted and concatenated before classification. (B) Multiple features are extracted, combined using an attention mechanism or neural network, and then classified. (C) A single feature extraction is performed, with an adapter network augmenting the features prior to classification. (D) Multiple features are extracted, concatenated, processed by an encoder, and then classified. The blue feature extraction blocks can entail predefined feature extraction, encoding, or both (like is indicated with the blue block in Fig. 9).

Methods that incorporate multiple features for classification, also termed early-fusion methods [378], usually employ one of four designs in the context of deepfake detection (Fig. 10). Most approaches extract different features and concatenate them before passing them to a classifier (Fig. 10A) [211,213,223,238,240,269,272,309,355,357]. Separate feature extraction branches are commonly used for different feature types such as spatial and frequency features [208,217,231,366], local and global features [282], chrominance and luminance features [207], temporal and spatial features [263], coarse-grained and fine-grained features [304] as well as different areas in the image such as facial regions [356] or features from different models such as CNN features and transformer features [302]. Another approach is to train an encoder in one branch on real images and another branch on fake images, making the overall feature vector capture features of both encoders [255]. Multimodal approaches have shown that combining images, text, video, and audio information in this way can improve performance as compared to using only unimodal features [244,272,293,328,329,340]. For videos, this approach has been used to concatenate features extracted from various frames [261,299,315,345].

In a second design, features are fused using more complex mechanisms like an attention layer [212,217] or a hierarchical fusion mechanism [218] (Fig. 10B). This design has mostly been used for incorporating local and global features [214,282,293,318,326,379], but also for combining video and audio information [293], ViT and CNN features [218] as well image and residual embeddings [227]. While more computationally expensive, experiments have shown that attention fusion can result in superior performance compared to simple concatenation [217].

In a third design, extracted features are concatenated with a processed version of those features modified by an adapter network [335] (Fig. 10C). This approach has been taken in cases where features need to be refined, but not fully transformed. For instance, Baru et al. used this strategy to refine the low-frequency components of CLIP embeddings [248]. Lastly, features can be concatenated and subsequently encoded into an embedding before classification [210,219] (Fig. 10D).

Other, more complex approaches have also been proposed. Feature fusion can occur multiple times during processing, for instance, when features from intermediate layers are concatenated and used in subsequent layers [205,206,304,355]. Alternatively, features can be combined in different ways. As an example, Chen et al. proposed an image realism score based on the size of a pentagon area composed of 5 image features [215].

#### 4.3.3 Ensemble models

**Fig. 11:**
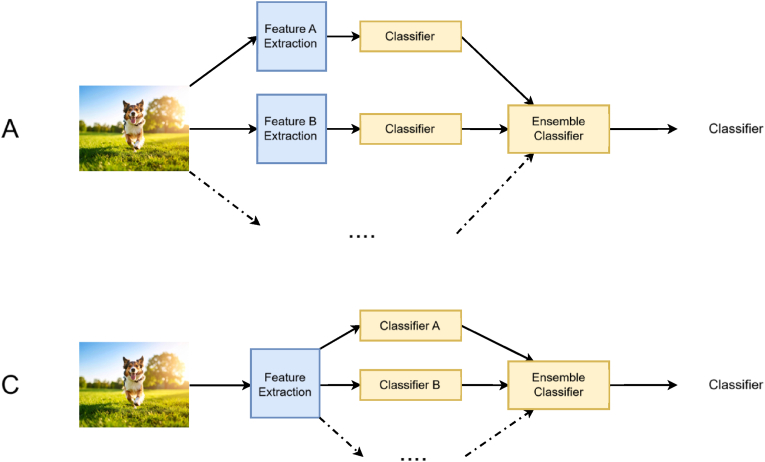
Types of ensemble models commonly used in the literature. (A) Multi-Feature Ensemble: different features are extracted from the image and each feature set is classified separately before combining results in an ensemble classifier. (B) Single-Feature Ensemble: A single feature extraction is performed and the resulting feature set is passed to multiple classifiers whose outputs are then combined in an ensemble classifier.

A last category of approaches leverages multiple models for detection, also termed late-fusion approaches [378]. In a first design, multiple models each classify based on different features and classifications are combined in an ensemble (Fig. 11A). In practice, this strategy is often used with different image patches that are each passed to a separate model [242,313,348,369,373]. Facial representation textures and a geometric representation distribution have also been integrated using this strategy [201].

A second approach is to train multiple different models on the same input features and classify with each of the models (Fig. 11B). This strategy has been used to combine the strengths of many different models [292], but also to combine predictions for models that have each been trained to detect a specific model family, the idea being that many specialists together perform better than a single generalist [274,380].

Most methods combine predictions from different models with mechanisms such as the weighted average score [201,224,250,381], possibly calibrating the scores from individual models [278]. Other approaches have trained a dense layer to attribute weights to various model predictions in a gated classification mechanism [276].

### 4.4 Training strategies

Many methods try to optimize their training strategy to achieve the highest performance and improve robustness and generalization. One of the most commonly employed strategies is to use data augmentation. Simple operations like rotation, scaling, zooming, grayscale conversion and compression have been widely used and have been shown to improve robustness and generalization [239,262,298,302,360]. More advanced approaches use artificial artifact generators applied to real and/or synthetic images during training [290,314], mask parts of the spatial or frequency domain [382], apply postprocessing enhancements such as super-resolution [221], or simulate GAN artifacts from various models [290] to make detectors more robust. Other methods have tried to reverse the effects of postprocessing operations instead [234].

Improvements to the optimization strategy itself have been proposed as well, for instance by using custom loss functions that integrate edge graphs or human saliency and thereby mitigate overfitting [216,284]. Furthermore, improved learning rate schedulers [306], meta-learning techniques [318] and genetic algorithms for optimization [213] can all have a beneficial effect on performance as well. The student-teacher learning paradigm has also been shown to improve generalization as it compresses the knowledge from a teacher model into a more condensed student model and thereby mitigates overfitting [289,337]. Others incorporate the detection model in a CICD pipeline or as a discriminator in a GAN framework, thereby ensuring continuous retraining of the detection model to reflect the latest generations [109,270,288,364]. In addition, evolutionary algorithms have been used to optimize the architecture of the model itself [383].

A separate set of strategies has been experimented with to improve vision language models. Multiple papers propose to finetune the CLIP encoder by incorporating an integration of shared and separate LoRAs as adaptable low-rank experts [333,342,377]. Finetuning in combination with real or fake class labels in the captions has also been shown to result in improved generalization performance [344]. Simple labels have been used, but including more concrete cues for deepfake detection has also shown promising results [376]. Lastly, prompt-tuning is a common strategy, where the text and image encoder of a vision language model are kept frozen and the learnable context around the text prompt embedding is optimized during training to adapt the model for the deepfake detection task [331,335,346,347,376].

Other approaches try to make training more effective and efficient by doing smart sample selection. For instance, by using Wasserstein clustering to compress the training dataset [334] or by evaluating samples using a quality metric [285]. Pairing real and fake images that belong to the same semantic category has been used to prevent semantic biases in the model [339,384].

### 4.5 Evasion

While less focused on overall in the literature, several studies also experiment with potential adversarial attacks or evasion strategies aiming to evade detection and mislead detectors. While the specific evasion methods and performance can differ depending on the detector and the input provided, overall evasion is relatively easy to implement and is generally quite effective. Even simple methods like removing characteristic frequencies present in generated images has been shown to already evade detection quite well [385,386]. Simple perturbations such as the addition of noise, shifting exposure, adding blur, or even adding a mix of artifacts of other generators can also distort characteristic traces and aid in evading detection [387,388]. Furthermore, innocent transformations like compression or application of super-resolution networks to upscale images have been shown to be effective methods to evade detection [389]. Making small additions in the latent space of generated images has also been shown to reduce the chance of detection, while only altering the semantic content of the image slightly [390]. Although such techniques are not always easy to apply without significantly visually altering the media content, networks can be trained to optimize the combination in which these perturbations are applied, such that there is virtually no difference from the original image [387].

Several methods also train a network to transform the frequency spectrum of an image to a seemingly natural frequency spectrum, causing the characteristic artifacts to be removed and detection largely evaded [87,391,392]. A GAN-like architecture can also be used where the discriminator spots artifacts which are removed by the generator [393] or where collaborative filtering is applied and the image is subsequently processed by a GAN generator [394]. If the attacker has access to the detection model in some way, the specific model can also be used to find adversarial examples or weak spots that evade detection, regardless of whether the attacker has access to the inner workings of the model or can only interact with it [395].

To make detectors more resistant against evasion strategies, a diverse set of adversarial examples should be included in the training set of detection models [395]. In addition, the generalization performance of detectors should be improved so they will be more flexible and can better detect media that fall outside the training distribution.

## 5 Evaluation

In this section, we will look into how deepfake detectors are evaluated and what general trends can be observed in evaluation across the literature. To this end, we will first look at what type of content most detectors are trained on to detect. We will subsequently give an overview of the most commonly used benchmark datasets in the field and discuss some general trends in the evaluation and performance of detection models.

### 5.1 Media types and model families

More papers are being published on synthetic media generation and detection every year. Interestingly, almost all detection publications we found are on synthetic images (98%) as opposed to videos (2%), whereas for generation this is more balanced with about 68% of publications focusing on images and 32% on videos. Detection publications for video have been published a lot, but focus almost always on manipulated media, leaving behind a gap in detection solutions that is otherwise difficult to spot.

**Fig. 12:**
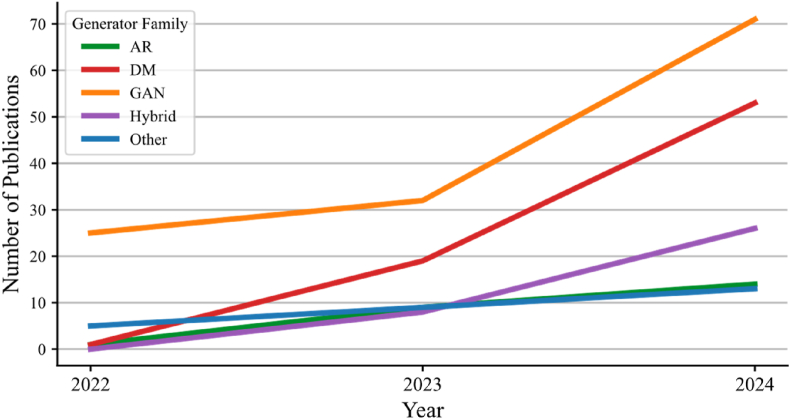
The number of generators considered in detection publications per year per model family.

**Fig. 13:**
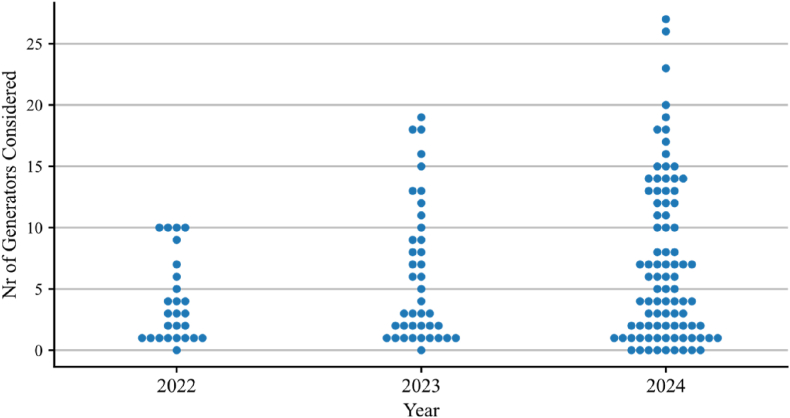
The number of generators considered by individual publications over the years. Every dot is a publication.

Fig. 12 shows the number of detection publications per year per model family that includes a certain generator in the training or evaluation set. GANs are clearly the most common type of generative model considered in the literature, although in recent years, DMs have been considered in a substantial part of the literature as well. While initially detectors were only evaluated on GANs, with the rise of DMs, these were included alongside GANs to evaluate generalization performance across model families. Other types of models have been considered slightly more over the years, but are considered much less often in general. Fig. 13 shows the number of different generators that are considered in publications over the years. A trend emerges here that shows an average increase in the number of generators considered per publication. This suggests that detectors train and evaluate on more diverse datasets as time progresses, incorporating a larger set of generative architectures. However, the distribution stretches out and does not completely shift, indicating that a substantial number of publications also still just considers only a few generators.

#### 5.1.1 Datasets

Table 1 shows an overview of the main benchmark datasets that are used for synthetic media detection. Most datasets have focused on synthetic images and provide substantial numbers of images from multiple generators. Datasets primarily include faces or general object categories as real images are almost always sourced from large real image datasets such as CelebA [414], CelebA-HQ [415], LSUN [416], LAION [417], ImageNet [267], MSCOCO [418], Flickr30k [419] and FFHQ [30]. Only a few fully synthetic video benchmark datasets have been published, containing generators trained on real video datasets such as Kinetics-400 [420], Youku-mPLUG [421], and MSR-VTT [422]. Real and synthetic samples are generally of high quality, and datasets contain only a limited variety of resolutions and file formats. While the images and videos in these datasets are representative for a lot of real world content, they do not necessarily represent the material seen during forensic casework, which is often of degraded quality, has bad lighting, or offset angles. In addition, many benchmark datasets preserve biases in generated images in aspects such as image resolution, file type, and semantic content. These biases have been shown to transfer to trained models and harm generalization performance [281]. A model like StyleGAN, for instance, has mostly been used for generating synthetic faces of a fixed resolution and is more likely to be predicted for images matching those details. Even though many synthetic image benchmark datasets have been released over the years, none of them seems to have established itself as the benchmark for synthetic image detection in the field. Many publications use different benchmark datasets, take subsets, or make their own dataset, the consequence being that results are still difficult to compare between publications.

**Table 1:** Benchmark datasets for synthetic image and video detection. Only generators used for full synthesis are included.

Dataset	Year	Semantic content	# Real	# Generated	GAN	DM	AR	Hybrid	Other
**Image**									
FakeSpotter [396]	2020	Faces	10.000	10.000	2				
iFakeFaceDB [397]	2020	Faces	-	87.000	1				
DFFD [398]	2020	Faces	58.703	300.000	2				
140K Real and Fake Faces [399]	2020	Faces	70.000	70.000	1				
ForenSynths [400]	2020	Objects	-	871.600	6				4
Hybrid Fake Face Dataset [401]	2021	Faces	60.000	95.000	2				
DiffusionDB [402]	2022	Varied	-	14M		1			
Deepfake Images Detection and Reconstruction Challenge [308]	2022	Faces	10.000	5.000	2				
Synthbuster [237]	2023	Varied	1.000	9.000		6			
ArtiFact [403]	2023	Varied	964.989	1.531.749	13	7			5
Continual Deepfake Detection Benchmark (CDDB) [404]	2023	Varied	-	1.2M	6				8
High Quality Deepfake Image (DFIM-HQ) [316]	2023	Faces	140.000	140.000	1				
GenImage [405]	2023	Varied	1.3M	1.3M	1	6		1	
UniFD [330]	2023	Varied	19.000	19.000	6	3	1		4
Fake2M [343]	2023	Varied	1.2M	2.3M	1	4	1		
DeepFakeFace (DFF) [406]	2023	Faces	30.000	30.000		1			
AntiFakePrompt [407]	2023	Varied	93.000	90.000		10			
DiffusionForensics [259]	2023	Varied	232.000	232.000		8			
DF40 [408]	2024	Varied	-	482.000	3	5		3	
GANGen-Detection [409]	2024	Faces	9.000	9.000	9				
WildRF [410]	2024	Social Media	2.650	2.650					?
DE-FAKE [340]	2023	Varied	59.247	59.247		4			
DeepFaceGen [411]	2024	Faces	350.264	350.264	1	9	1	1	
SAMGAN3 [302]	2024	Faces	39.000	39.000	3				
CIFAKE [367]	2024	Varied	60.000	60.000		1			
Diffusion-generated Deepfake Detection dataset (D3) [412]	2024	Varied	2.3M	9.2M		4			
COCO-FAKE [336]	2025	Varied	-	1.2M		2			
DeepGuard [296]	2025	Varied	6.000	8.650		3		1	
TwinSynths [348]	2025	Varied	-	16.000	1	1			
**Video**									
CMDFD [371]	2024	Faces	1.000	8.000					2
DeepFaceGen [411]	2024	Face	423.548	423.548		5			
GenVideo [345]	2024	Varied	10.000	2.3M		12		2	5
MSTF [359]	2024	Faces	37.059	106.695					5
GenVidBench [413]	2025	Varied	33.931	108.000		5	1		2

**Fig. 14:**
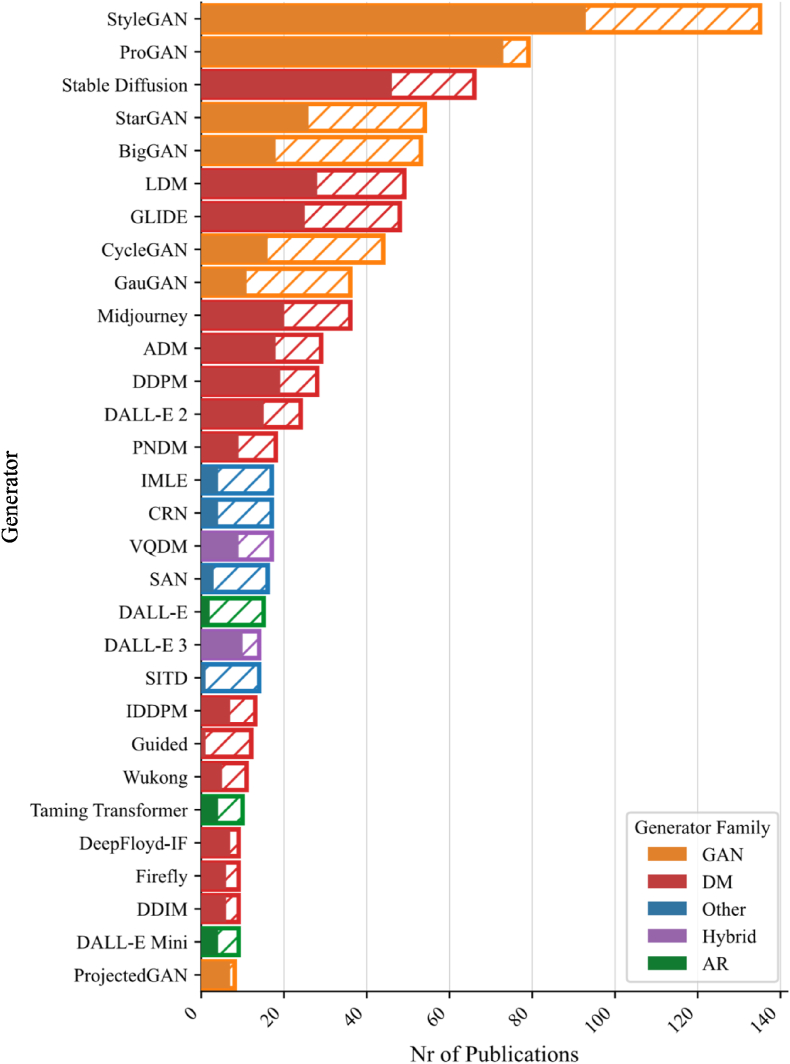
The top 30 generators considered by publications from 2022 to 2025 for the development of synthetic image detectors. The criterion to include a generator as considered by a publication is that it was used for training, evaluation or both. The filled part of each bar indicates the number of publications that used this particular generator for both training and testing. The remaining dashed part of the bar indicates the publications that only evaluated on this generator but trained on different generators. Different versions of the same architecture have been grouped.

A large variety of generators is considered across the literature. Fig. 14 shows the top 30 generators that are considered for the development of synthetic image detectors. The solid filled part of the bar indicates the number of publications that include a generator in the training or evaluation set, whether the publications in the dashed part only include the generator in the evaluation set. Generators like StyleGAN and ProGAN have been considered quite often, given their fundamental role in the field and the widespread availability of datasets for these generators. Especially, ProGAN is used relatively often for training, sometimes in combination with LDM. GANs seem to be considered most often, followed by DMs. As mentioned before, this is a result of the practice that DMs are commonly included besides GANs in evaluation sets nowadays to evaluate generalization across model families. Interesting is that commercial tools such as Adobe Firefly and DALL-E 3 have been considered much less often, even though they are more accessible to the general public. This could be due to the fact that it is relatively impractical to include such generators in datasets, as they often cost money to use, have limitations on the generation rate, and do not run locally. As a result, these commercial generators are also mostly only used for evaluation to assess generalization performance, but are rarely included during training.

### 5.2 Evaluation criteria & performance

Essentially all methods in the literature produce a classification and are therefore commonly evaluated using classification metrics like accuracy, precision and recall. Most methods aim at predicting binary labels (e.g. real or fake), although some also predict model family labels (e.g. GAN, DM) or even specific generator architectures (e.g. StyleGAN2, LDM, etc.) [315]. Multi-level ensemble approaches aiming to predict labels at increasing levels of detail have also been proposed, the idea being that different traces might be useful for differentiating at different levels [214,268,286,296,366]. By training, different models per level, the responsibilities of each model are more specific, leading to improved performance. Some have also used other labels, for instance for compressed images [247], authentic images that were modified using generative AI models [423], or images that had been processed by multiple generation cycles [245].

SOTA detectors achieve near-perfect accuracy on in-distribution samples, but struggle with out-of-distribution generalization, reporting accuracies between 70% and 100% for unseen generators. Inconsistent evaluation protocols, including heterogeneous datasets and generator selections, complicate comparisons between studies. Generalization performance is commonly evaluated by training on a single generator such as ProGAN [29] or LDM [2] and testing generalization across a diverse set of architectures. Results are inconsistent on which families to include in the training set. Training on diffusion models (DMs) may mitigate overfitting to generator family-specific artifacts more prominent in GANs [424], but training on GANs has demonstrated superior generalization performance in other studies as well [425]. Hybrid approaches combining GANs and DMs for training generally achieve good results, although research has also shown that training on too many generators harms generalization performance [377]. Results are consistent on cross-domain evaluations, showing that training on a more diverse set of semantic categories does result in improved cross-domain generalization performance [297,350]. While larger datasets may enhance performance for generators sharing similar artifact patterns, they may also induce overfitting, thereby reducing generalization capabilities [254].

Neural network embeddings have been used for the detection of many different generators. Embeddings from vision transformers (ViTs) in particular, seem to outperform convolutional networks (CNNs) in cross-generator generalization [305], likely due to their global attention mechanisms. Prompt tuning and contrastive learning strategies with these models further boost generalization. Semantic features have mostly been explored for GAN-based architectures such as StyleGAN, while reconstruction error-based approaches have experimented more on DM models. Feature fusion strategies integrating multiple features generally seem to obtain improved detection accuracy compared to single-feature models, although the added complexity may not always be worth it for slight performance improvements.

Approximately 20% of studies evaluate robustness to post-processing, primarily testing JPEG compression and blurring. Less common perturbations include noise addition, rotation, and resizing. Current detectors show limited resilience, maintaining performance under mild perturbations but degrading significantly with intensity. Robustness to perturbations therefore remains a challenge in synthetic media detection.

## 6 Explainability

In forensic practice, explainability and interpretability of methods is of vital importance to ensure experts can rely on detection methods for their conclusions in court. For many methods using explainable features in combination with simple classifiers, explainability is not an issue. However, many SOTA methods employ deep learning models, either for feature extraction, classification or both.

For CNN classifiers, classic visualization techniques such as Grad-CAM [426] can be used to explain classifications [317,427], while for transformers the attention maps may be visualized. While these techniques give some insight into image areas that influence a model’s prediction, actual explanations of why these areas are important are still not provided. Insertion or deletion techniques such as SHapley Additive exPlanations [428] or LIME [429] have been used as well [427], but are generally not well suited for the deepfake detection task as they might introduce new artifacts leading to inconsistent results [430]. In a subjective evaluation experiment of various model explanations, Yang et al. [321] found that people generally prefer explanations given by rollout [431] as these explanations generally correspond better to seemingly relevant features compared to techniques like Grad-CAM [426], layerwise relevance propagation [432] and transformer attribution [433]. However, visual explanations alone are usually still not enough to understand the reasons behind a prediction. Furthermore, visual explanations may not always be the most relevant.

Prototyping approaches have implemented increased explainability of deep learning features as well, for instance by showing the prototype samples most similar to a questioned sample [325,434]. While such techniques give insight into how a sample is positioned in the feature space of the model, the decision criteria remain opaque and samples shown are very dependent on the prototypes in the prototype set. In addition, explainable AI techniques may also bias the human user, by focusing on some aspects of the input while ignoring others. Hence, this is an important element to consider when designing these explainable AI techniques.

## 7 Open challenges and future research

Although research on synthetic media generation and detection is advancing rapidly, several open challenges persist and remain active areas of investigation. In this section, we will discuss some interesting directions for future research based on current challenges in the field and future opportunities for applications.

### 7.1 Generalizability to unseen generators

As discussed in Section 5, a primary challenge in the current literature is the generalizability of detection methods to unseen generators. This issue is especially relevant in forensic contexts, where the specific generator responsible for a questioned image is almost always unknown. Although numerous methods claim improvements in generalizability, direct comparisons are hindered by heterogeneous evaluation datasets, resulting in conflicting findings. However, the development of detectors that generalize well should be an important focus for future research.

### 7.2 Robustness to perturbations

Another significant challenge is the robustness of detection methods to common image perturbations such as compression, rotation, cropping, and blurring. In section 5 we showed that only a limited set of studies assesses robustness. Furthermore, the perturbations examined do not always align with those encountered in forensic practice. For example, while compression, blurring, and noise addition are frequently studied, real-world perturbations like screenshotting are often overlooked. Moreover, synthetic augmentation of perturbations may not capture the diversity of real-world perturbations [410]. In addition, many modern cameras also incorporate more advanced forms of postprocessing, often using AI-based algorithms. Such postprocessing may introduce traces into natural images as well, making it more difficult to distinguish synthesized media from camera-captured media. Future research should therefore focus on evaluating and enhancing resistance to such perturbations, incorporating real-world data to improve robustness [297]. Additionally, as we discussed in section 4.5, robustness against adversarial attacks requires further investigation.

### 7.3 Standardization and benchmarking

Despite the existence of many published benchmark datasets, we observed that evaluation protocols vary widely, with relatively few studies employing identical datasets or benchmarks. This inconsistency complicates benchmarking and comparative analysis. As the field moves rapidly, new benchmark datasets should continue to be developed. However, existing benchmark datasets should be used alongside these new benchmarks to facilitate comparison with existing methods.

### 7.4 Lack of synthetic video detectors

We also saw in section 5 that while numerous methods target synthetic image detection, relatively few address fully synthetic video detection. Many existing video detection studies focus on manipulated videos or include only synthetic images [299,372]. Concurrently, the active development of synthetic video generation architectures underscores the urgent need for dedicated synthetic video detectors to keep pace with generation capabilities.

### 7.5 Mismatch between academic research and practical casework

There is a notable discrepancy between generators commonly used in academic evaluations and those prevalent in practical forensic casework. Additionally, evaluation datasets often contain images of lower resolution than those encountered in real cases. Furthermore, the effects of chaining multiple generators, where outputs from one generator serve as inputs to another, on detection remain insufficiently studied. These factors together suggest that academic evaluations may not fully reflect real-world performance. Future detection methods should strive to bridge this gap by incorporating data representative of forensic casework.

### 7.6 Potentially unexplored detection methods

Several detection techniques developed for manipulated media, such as those based on electrical network frequency (ENF) analysis [435] and analysis of blood flow in the face [14,436], have demonstrated promise but remain underexplored for fully synthetic media, particularly synthetic video. These approaches would be interesting potential avenues for advancing synthetic media detection.

### 7.7 Explainability

In section 6 we discussed that detection methods should be explainable to a certain extent to be useful in forensic casework. Although deep neural networks achieve high accuracy in synthetic image detection, their black box nature limits interpretability at the case level. Existing explainable AI approaches still offer limited insight into individual decisions and future work should therefore prioritize explainability more.

### 7.8 Adoption of the likelihood ratio framework

All detection methods we discussed output classification scores or labels, whose uncertainty is often difficult to interpret (e.g., what does a 70% synthetic score imply?). Moreover, image authenticity depends on contextual factors, such as the ease with which a suspect could have generated an image, which are not taken into account by most detection methods. The likelihood ratio (LR) framework has therefore been adopted in many countries as the standard for reporting evaluative evidence. Machine learning models can be adapted to calculate LRs instead of confidence scores relatively easily [437]. Future research should therefore strive to also calculate LRs in order to facilitate the adoption of deepfake detection methods in forensic practice.

## 8 Conclusion

The field of synthetic media generation and detection is moving rapidly. Research on generating images and videos using AI has focused primarily on improvements in visual fidelity, fine-grained user control, and computational efficiency. The primary focus of detection research remains on enhancing the generalizability and robustness of AI-generated image detectors. Despite the publication of numerous benchmark datasets for synthetic image detection, detection methods remain difficult to compare due to the inconsistent evaluation protocols applied. Moreover, the forensic relevance of existing benchmarks is limited, where real-world generators are only taken into account to a limited extent, and the perturbations considered do not always reflect those encountered in practical forensic contexts. To further advance the field, future research on deepfake detection in the forensic context should encourage the development and adoption of benchmark datasets that accurately reflect forensic casework conditions. Furthermore, the explainability of the detection methods should be prioritized more and the methods should be integrated into the likelihood ratio framework, thus increasing the applicability of these methods in forensic casework.

## Declaration of competing interest

The authors declare that they have no known competing financial interests or personal relationships that could have appeared to influence the work reported in this paper.

## Appendix A Advanced Keyword Search Queries

These are the advanced keyword queries used for the initial systematic literature search per library.

### Appendix A.1 Scopus

TITLE-ABS-KEY ( ( "deepfake" OR "deep fake" OR "synthetic media".

) AND ("image" OR "video" OR "picture") AND ( "detection" OR "identification".

OR "recognition" OR "classification" OR "analysis" OR "forensics".

OR "generation" OR "creation" OR "synthesis") ) AND PUBYEAR >2021.

AND (LIMIT-TO ( DOCTYPE, "cp") OR LIMIT-TO (DOCTYPE, "ar").

) AND (LIMIT-TO ( SUBJAREA, "MULT") OR LIMIT-TO ( SUBJAREA, "COMP".

) OR LIMIT-TO (SUBJAREA, "ENGI") OR LIMIT-TO ( SUBJAREA, "MATH".

)) AND (LIMIT-TO ( LANGUAGE, "English") ).

### Appendix A.2 IEEE Digital Library

("All Metadata":"deepfake" OR "All Metadata":"deep fake" OR "All.

Metadata":"synthetic media") AND ("All Metadata":"image" OR "All.

Metadata":"video" OR "All Metadata":"picture") AND ("All Metadata":"detection".

OR "All Metadata":"identification" OR "All Metadata":"recognition" OR "All Metadata":"classification" OR "All Metadata":"analysis" OR "All Metadata":"forensics" OR "All Metadata":"generation" OR "All Metadata":"creation" OR "All Metadata":"synthesis") Conference and journal filters applied. 2022-2025.

### Appendix A.3 ACM Digital Library

[[All: "deepfake"] OR [All: "deep fake"] OR [All: "synthetic media"]] AND [[All: "detection"] OR [All: "identification"] OR [All: "recognition"] OR [All: "classification"] OR [All: "analysis"] OR [All: "forensics"] OR [All: "generation"] OR [All: "creation"]

OR [All: "synthesis"]] AND [[All: "image"] OR [All: "video"] OR [All: "picture"]] AND [E-Publication Date: (01/01/2022 TO ∗)] Filtered on proceedings, journal publications and research articles.

## Footnotes

4 https://www.scimagojr.com/journalrank.php.

5 http://www.conferenceranks.com/.

6 https://scie.lcc.uma.es/.

## Footnotes

1 https://www.scopus.com/pages/home.

2 https://ieeexplore.ieee.org/Xplore/home.jsp.

3 https://dl.acm.org/.
